# Intimate partner violence associated with low quality of life - a cross-sectional study

**DOI:** 10.1186/s12905-018-0638-5

**Published:** 2018-09-04

**Authors:** Kjersti Alsaker, Bente E. Moen, Tone Morken, Valborg Baste

**Affiliations:** 1 Faculty of Health and Social Sciences/ Department of Welfare and Participation, Western Norway University of Applied Sciences, Haugeveien 28, 5005 Bergen, Norway; 2 National Centre for Emergency Primary Health Care, Uni Research Health, Bergen, Norway; 3 Centre for International Health, Department of Global Public Health and Primary Care, University of Bergen, Bergen, Norway; 4 Uni Research Health, Bergen, Norway

**Keywords:** Intimate partner violence, Abuse, Quality of life

## Abstract

**Background:**

Quality of life among abused women in Norway in 2006 was found to be significantly low compared to women at the same age in general. The aim of this study was to examine how quality of life is associated with experience of psychological and physical violence intimate partner violence among abused women seeking help after domestic partner abuse comparted to quality of life in a random sample of women in Norway.

**Methods:**

A cross-sectional study in a random sample of 1500 women (response rate 36%, *n* = 469) in Norway were performed. In addition, 191 women who sought help after domestic partner abuse were invited (44%, *n* = 84). The experience of intimate partner violence (IPV) and health-related quality of life were measured in both samples. The participants were divided into: “Women seeking help” after domestic partner abuse (*n* = 84); “Random sample, abused women” (*n* = 127); and “Random sample, not abused women” (*n* = 342).

**Results:**

The experience of psychological and physical violence was significantly different between the groups (*p* <  0.0001). The domains in SF-12 were significantly below (*p* <  0.001) the norm for the female population in Norway in all dimensions among the abused women in the random population sample, and even lower among the women seeking help because of IPV.

**Conclusion:**

Intimate partner violence is clearly associated with low quality of life. The pattern found in this study is similar to the pattern found in the previous Norwegian study among abused women seeking help.

## Background

Intimate partner violence (IPV) against women is a major health problem worldwide, with a serious impact on women’s physical and mental health [1–3]. IPV refers to any behaviour within an intimate relationship that causes physical, psychological or sexual harm to those in the relationship [4]. A Norwegian study found a 27% lifetime prevalence of physical IPV against women, and 9% of these had experienced serious physical violence [5]. This has been referred to as a Nordic Paradox because the level of gender equality in Norway and other Nordic countries is high [6]. In a study about quality of life among abused women in Norwegian women’s shelters in 2006, the reported health-related quality of life (HRQoL) was seriously low (scores 20–32) on mental health, vitality and social function just after arriving at a women’s shelter [7]. The very low HRQoL reported was important to establishing a focus on this issue in health and social services in Norway [8]. Quality of life is found to be significantly low among abused women around the world [9, 10]. When the mental health score drops from 40 to 20, suicidal ideation increases by 105% [11]. Femicide and suicide are the most serious consequences of IPV against women [3, 12–14].

The aim of this study was to examine how quality of life is associated with experience of both psychological and physical violence intimate partner violence (IPV) among abuses women seeking help from Police, Women’s shelters, Alternative to Violence, Assault Centre and/or Family Guidance Centers compared to quality of life in a random population sample of women in Norway.

Knowledge about psychical, social and mental health problems, is useful in assessing the need for health care among abused women.

## Methods

### Participants and study design

A cross-sectional study was performed. The questionnaire was available in both Norwegian and English to make it possible to include women with other ethnic backgrounds than Norwegian. We included two samples: The first was “Abused-Seeking help”: The questionnaire was sent to institutions where women may seek help after domestic partner abuse, such as the Police, Women’s shelters, Alternative to Violence, Assault Centre and Family Guidance Centers. Inclusion criteria among the women seeking help were experience of partner abuse and understanding (reading) Norwegian or English. The second sample was a random sample from the population: The questionnaire was sent to a random sample of women, picked and sent by Statistics Norway. One reminder was sent to every recipient. Inclusion criteria in the random population sample were being a woman between 18 and 70 years; living in Hordaland, Norway; had been living with a partner and understanding Norwegian or English.

### Psychological and physical violence

The questionnaire consisted of questions about demographics and degrees of psychological and physical violence, as well as health-related quality of life (HRQoL). Psychological violence was measured by the Psychological Maltreatment of Women Inventory (PMWI) short form [15], containing 14 questions with five respons categories (Never, Rarely, Occasionally, Frequently and Very frequently).Physical violence was measured by the Norwegian measurement used in population studies [5], containing 12 questions with respons category yes or no, regarding both last year and before last year.

### Health-related quality of life

HRQoL information was gathered using the SF-12 health survey, which consists of 12 items divided into eight scales. The SF12 is derived from the SF-36 health survey, which is one of the most widely used generic instruments to measure physical and mental health-related functioning [16]. SF-12 is tested for validity and reliability, and also tested against the SF-36 [16–19]. The scales include physical functioning, physical role, bodily pain, general health, vitality, emotional role, social functioning and mental health. Raw scores for each scale range from 0 to 100, and adjusted median scores from 0 to 50, with lower scores reflecting poorer functioning. In this study, the standard Norwegian version was used, which asks about health situations in the past four weeks. The results from SF-12 were adjusted for age according to the general female population, such that the mean of the general population is 50 and the standard deviation is 10.

### Analysis

Only those who had been living with a partner were included in the analyses. Psychological violence was dichotomized into no (never/rarely/occasionally) or yes (frequently/very frequently). Physical violence was categorized into yes or no, regardless of if the physical violence was experienced last year or before last year. The data were analyzed in three groups “Women seeking help” after domestic partner abuse, and “Random sample, abused women” and “Random sample, not abused women”.

We used mean and standard deviation (SD) to examine years of age and education in the three groups. Occurrence of different acts of physical and psychological violence was given for “Women seeking help” and “Random sample, abused women” and differences between the two groups were tested in chi square analysis. To compare mean differences in HRQoL between the three group analyses of variance (ANOVA) were used. The Bonferroni were used in the post hoc tests in the ANOVA, to adjust for multiple testing. We also compared HRQoL in the random sample of with normative adjusted data from the general population in Norway by t-test (Fig. 2). Cronbach’s alpha was used to test reliability among of the dimensions of SF-12 in this study. Cronbach’s alpha in the eight standardized items in this study in SF-12 varied from 0.82 in the women seeking help sample to 0.89 in the random sample of abused and not abused women. In the total sample (*n* = 551) Cronbach’s alpha was 0.92.The data were analyzed using IBM SPSS Statistics 23 for Windows.

### Ethical considerations

The study was approved by the Regional Committee for Medical and Health Research Ethics.

The questionnaire does not include personal data as name, date of birth or address and are coded anonymously. The first page in the questionnaire gave information about the study and the right to decline to answer. We also included telephone number to the researcher. Completion and return of the questionnaire was seen as consent to participate in the study.

## Results

In this study 551 women participated; “Women seeking help” (*n* = 82); “Random sample” (*n* = 469). The response rate was 44% in the sample of “Women seeking help” and 36% in the random population sample. Thirteen people (2.7%) answered in English. The women seeking help were significantly younger than the women in the population sample (Table 1). No significant difference in education was found. Physical and psychological violence were more frequently reported in the group of women seeking help than in the random population sample of abused women regarding all acts (*p* <  0.001) (Table 2).

**Table 1:** Sociodemographic characteristics: “women seeking help because of partner-abuse” and “random sample of abused women” in Norway

	Seeking help (*n* = 82)		Random sample (*n* = 469)				
	Abused		Abused		Non-Abused		
	Mean	(SD)	Mean	(SD)	Mean	(SD)	*p*-value^1^
Age	38.4	(11.1)	42.9	(13.6)	45.5	(13.1)	< 0.001
Year of education after primary school	4.8	(3.2)	5.5	(3.1)	5.4	(3.0)	0.238
Psychological violence V/E	28.6	(7.2)	15.0	(6.7)	7.9	(1.7)	< 0.001
Psychological violence D/I	25.2	(8.7)	12.1	(6.4)	7.5	(1.0)	< 0.001

^1^From ANOVA

**Table 2:** Physical and psychological violence among “women seeking help because of partner-abuse” and “random sample of abused women” in Norway

Physical and psychological violence	Seeking help		Random sample	
	Abused		Abused	
	*n*	%	*n*	%
Physical violence during the last 12 months, and/or at any time in your life before that				
Threatened to hurt you or others you are found of?	65	81.3	36	29.0
Threatened to kill you?	53	67.1	22	17.6
Obstructed you from moving around freely, or grabbed and hold you with force?	65	82.3	61	48.8
Hit you with an open hand?	53	67.1	46	36.8
Threw a hard object at you?	45	57.0	33	26.4
Hit you with a clenched fist, a hard object or kicked you?	55	68.8	28	22.6
Had a stranglehold or tried to strangle you?	44	55.7	21	16.8
Assaulted you with a knife or other type of weapon?	28	35.4	8	6.4
Hit your head against an object or against the wall or the floor?	40	50.0	13	10.4
Forced you to have sex against your will?	49	63.6	28	22.4
Behaved violent toward you in other way?	73	93.6	71	53.6
Repeatedly followed you, phoned or visited you at work so that you became afraid?	55	69.6	21	16.8
Psychological violence frequently or very frequently				
Called me names	62	77.5	20	15.9
Swore at me	57	72.2	17	13.6
Yelled and screamed at me	61	76.3	16	12.7
Threated me inferior	63	78.8	20	15.9
Monitored my time and demanded to know where I was	53	66.3	15	12.0
Used money or made important financial decisions without talking to me	49	61.3	16	12.7
Was jealous or suspicious of my friends	55	70.5	22	17.5
Accused me for having an affair with another	35	43.2	12	9.6
Interfered my relationship with other family members	35	67.9	12	9.5
Tried to keep me from doing things to help myself	43	55.8	8	6.4
Restricted my use of the telephone	33	41.3	8	6.5
Told me my feelings were irrational or crazy	61	76.3	19	15.2
Blamed me for his problems	61	76.3	24	19.4
Tried to make me feel crazy	59	73.8	13	10.5

The respondents reported serious physical and psychological violence. Threats of being hurt were reported by 81% among women seeking help, and 29% among the abused women in the random sample, further forced to have sex (64% versus 22%, respectively) and stranglehold (56% versus 17%) (Table 2). Acts of psychological violence as verbal/emotional abuse about calling her names, sworing, yelling and acts related to jealousy frequently/very frequently were reported by more than 70% in the “women seeking help” group. These acts were also the most frequently acts reported among the abused women in the random sample, however much lower (10–17.5%). Acts related to dominance and control as jealousy, monitoring her time, making important financial decision without talking to her showed the same patterns however it was less frequently reported.

**Fig. 1:**
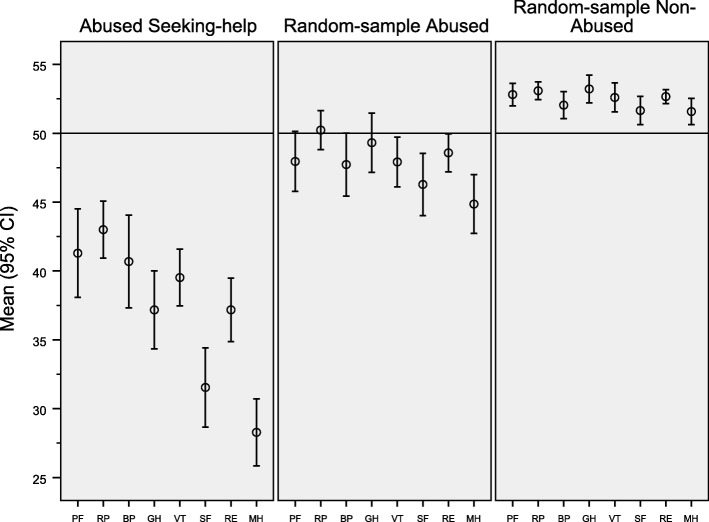
Mean age-standardized score and 95% confidence interval (CI) for the eight SF-12 dimensions among “ abused women seeking help”, “random sample of abused women” and “random sample of not abused women”. The mean score of the general Norwegian female population is 50 for all scales. (PF = Physical Function, RP = Role Physical, BP = Bodily Pain, GH = General Health, VT = Vitality, SF = Social Function, RE = Role Emotional, MH = Mental Health)

The SF-12 scores were significantly different among the groups (Fig. 1). All domains in SF-12 were significantly lower (*p* <  0.001) among the women seeking help after domestic partner abuse compared to abused women in the random sample. Further the abused women in the random sample had significant lower health related quality of life (SF12) compared to the not abused women in the random sample. The social functioning and the scores in the mental health domain were two standard divisions below the normal population (Fig. 1).

**Fig. 2:**
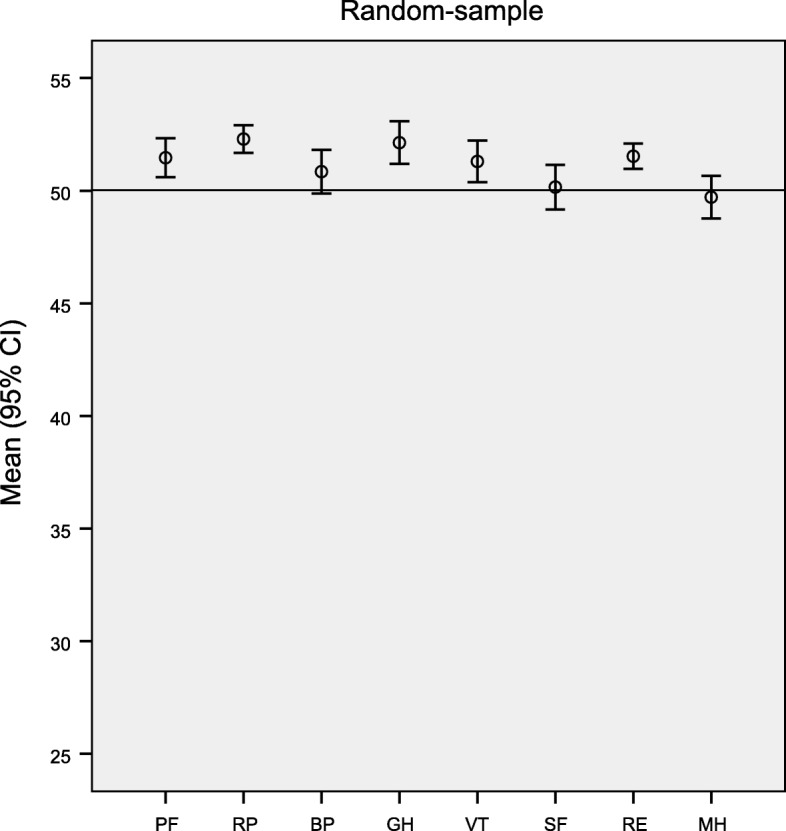
Mean age-standardized score and 95% confidence interval (CI) for the eight SF-12 dimensions in the total random sample of women. The mean score of the general Norwegian female population is 50 for all scales. (PF = Physical Function, RP = Role Physical, BP = Bodily Pain, GH = General Health, VT = Vitality, SF = Social Function, RE = Role Emotional, MH = Mental Health)

When comparing the random sample with the population norms in HRQoL in Norway, we found significantly higher scores (*p* < 0.01) in all domains except mental health, social functioning and bodily pain (Fig. 2).

## Discussion

Women who had experienced partner abuse reported lower HRQoL in all domains compared to women who had not experienced IPV. The social functioning and mental health scores were two standard divisions below the norm in population scores among women of the same age in Norway, as found in our earlier study [6]. These scores are very low, and indicate a risk of suicide [11]. The women seeking help after domestic partner abuse, also had especially low scores in mental health. New studies from Iran, China and USA confirm the significantly lower quality-of-life scores among abused compared to non-abused women [14, 19, 20].

Serious threats, such as threats to kill you or someone you care for, reported by more than two thirds of the sample of women seeking help and nearly one third in among abused women in the random sample, shows that these acts are valid related to IPV The relationship between IPV and quality of life, highlights the fact that violence destroy the quality of life in these women’s lives.

The very low mental health score found in our study in 2006 [6] is also found in the present study, and it is still lower than scores reported in other studies [19–22]. This may be an effect of feeling even more left out and alone when experiencing partner violence in a society where the level of gender equality is high. However, “gender equality” refers to social norms that may differ from personal norms in close relationships among partners. Many men may still experience a need to feel more powerful than women. These factors may be related to the Nordic Paradox [6].

The events that trigger and most of the acts of men’s violence against women are found to be remarkably consistent throughout the world [4]. Power and control strategies that keep women in the subordinate position is central. Some norms as “blaming the victim” and “keep your private problems private” make it difficult to tell about IPV problems. These norms have been slowly changing during the last decades and may change more rapidly in the wake of the Meetoo campaign. Shame and guilt are negative feelings and these emotions are strongly related to low quality of life. The deepest feeling of shame is defined as a strong feeling of not being worthy as a human being [23].

In our earlier one-year follow-up study among abused women in Norwegian women’s shelters, the quality of life was significantly improved with regard to vitality, social function and mental health among those who had left their partner, but their low physical health scores were not improved [24]. This may indicate that recovery from bodily effects of IPV requires more time and maybe intervention.

The higher scores in the general population sample in this study with regard to “physical health” “physical health role” and “role- emotional” may be related to the character of this study, as it was called “Work, health and safety survey” and includes a sample with higher employment rates and therefore also higher HRQoL scores. In addition, others have found that women experiencing IPV seldom answer questionnaires about IPV because they are afraid of reprisals from their husband. The more IPV experienced, the more infrequently they respond to such questionnaires [25].

The response rate was low, but this is common in these kinds of studies [25, 26] and may be a result of the very private and taboo nature of the questions asked. Strength of the study is the inclusion of both women seeking help and a normal population. As this study is a cross sectional study we can only conclude on associations. The reason why abused women have low quality of life cannot be stated by this study. The nature of the question may also influence the way of responding. In the population study, the participants answered the questions at home, and might be under influence of others. This may have led to different reporting from this group, compared to the answers from the abused women. However, we cannot state this with certainty. The high frequency of different acts of IPV reported by “women seeking help” confirm that women seeking help because of IPV must be prioritized, and danger assessment as well as safety planning must be done. Using SF-12 in measuring HRQoL among abused women provides an opportunity for comparing the results with the general female population and with other relevant studies abroad.

## Conclusion

Intimate partner violence is clearly associated with low quality of life. We found a pattern similar to the pattern found in the previous Norwegian study among women seeking help after domestic partner abuse. This highlight the need for actions to reduce the intimate violence against women and greater awareness among health professionals to address this issue.

## Abbreviations

HRQoL: Health-related quality of life
IPV: Intimate partner violence
